# Adsorption and Flame Retardant Properties of Bio-Based Phytic Acid on Wool Fabric

**DOI:** 10.3390/polym8040122

**Published:** 2016-04-05

**Authors:** Xian-Wei Cheng, Jin-Ping Guan, Guoqiang Chen, Xu-Hong Yang, Ren-Cheng Tang

**Affiliations:** National Engineering Laboratory for Modern Silk, College of Textile and Clothing Engineering, Soochow University, 199 Renai Road, Suzhou 215123, China; chengxian-wei@outlook.com (X.-W.C.); guanjinping@suda.edu.cn (J.-P.G.); chenguojiang@suda.edu.cn (G.C.)

**Keywords:** wool, phytic acid, adsorption, flame retardant, burning behavior

## Abstract

Bio-based phytic acid (PA) as a nontoxic naturally occurring compound is a promising prospect for flame-retardant (FR) modifications to polymers. In this work, PA was applied to wool fabric using an exhaustion technique, and the adsorption and FR properties of PA on wool fabric were studied. The flame retardancy of the treated wool fabrics depended greatly on the adsorption quantity of PA, which was related to the pH of treatment solution, immersing temperature and initial PA concentration. The Langmuir adsorption of PA took place due to electrostatic interactions between PA and wool fiber. The limiting oxygen index, vertical burning and pyrolysis combustion flow calorimetry tests revealed that the treated wool fabrics exhibited good flame retardancy. The measurements of the phosphorus content of the burned fabric residues and thermogravimetric analyses suggested that a significant condensed-phase FR action was applicable to the PA treated fabrics. PA treatment was found to have little adverse effect on the whiteness and mechanical performance of wool. Additionally, the washing resistance of the FR fabrics should be further improved.

## 1. Introduction

As a natural protein fiber, wool is widely used in apparel, interior textiles and industrial clothing for its comfort and a high level of inherent flame resistance. The inherent flame-resistant properties of wool are related to its relatively high nitrogen (15%–16%) and sulfur (3%–4%) content, high moisture content (10%–14%) and low heat of combustion [1,2]. However, most wool fabrics can pass a horizontal test but cannot pass 45° or vertical tests. Some special products such as seat coverings in cars and buses, aircraft furnishings and blankets, wall coverings in public buildings, protective clothing, and carpets of shag pile construction and low density need additional treatment to impart higher flame resistance [3].

The Zirpro flame retardant (FR) treatment, which can markedly improve the flame resistance of wool, are based on the exhaustion of negatively charged zirconium or titanium complexes onto positively charged wool under acid conditions [4,5]. The Zirpro treatment has become the most commonly used durable FR process for wool [6]. Based on the Zirpro process, many kinds of acids such as formic acid and hydrochloric acid have been applied along with zirconium or titanium complexes to improve the FR properties of wool fabrics [7,8,9,10]. Some intumescent agents such as tetrakis-hydroxymethyl phosphonium condensates (THPC), *N*-methylol dimethyl phosphonopropionamide derivatives (MDPA) and simple ammonium phosphates have also been applied to increase the FR properties of wool fabric by means of the formation of a high thermal resistance insulation char layer [11,12,13]. Other approaches have been used to enhance the FR properties of wool [14,15]. As a whole, though, little has changed since 1986, when Horrocks comprehensively discussed the development of flame retardants for wool [9,11].

Considering the cons of the flame retardants used, the USA and EU directives concerning the chemistry of flame retardants are becoming increasingly rigid and severe, so some of the currently used products will be limited or even banned. Therefore, both the academic and industrial communities should continuously invest time and funds in order to find worthy alternatives to traditional flame retardants. In this context, very recently, some biomacromolecules like proteins and nucleic acids have been thoroughly investigated because they exhibit significant potentials as novel green FRs for selected textiles [16]. For example, caseins, hydrophobins, whey proteins and deoxyribonucleic acid have been successfully applied to improve the FR properties of cotton fabrics by padding techniques [17,18,19,20]. In addition, caseins have been found to be efficient for the improvement of the FR capability of polyester and polyester/cotton blends [21].

Recently, a bio-based phosphorus-containing compound phytic acid (PA) has provoked people’s interest in textile treatment, especially in FR treatment. PA is known as inositol hexakisphosphate acid or phytate in the salt form, and regarded as a “green” molecule because it is found in abundance in plants tissues, such as beans, cereal grains and oil seeds [22,23]. As a biocompatible, environmentally friendly, nontoxic and easily obtained organic acid [24,25], PA has already been widely applied in antioxidant, anticancer, biosensor, cation exchange, nanomaterial and other fields because of its special inositol hexaphosphate structure [26]. PA contains 28 wt % phosphorus based upon molecular weight, and is promising as one of possible and effective FR materials. PA has been used to reduce the flammability of cellulose-based materials. PA has been employed as a doping acid to greatly improve the FR performance of polyaniline-deposited paper composite [27]. PA/chitosan and PA/nitrogen-modified silane hybrids have been used via layer-by-layer assembly to fabricate FR thin films on cotton fabric [26,28]. In addition, the potential FR effect of different metallic phytates has been evaluated as bio-sourced phosphorus additives for poly(lactic acid) composites [29].

PA consists of six negatively charged phosphate groups and has a strong tendency to combine or interact with positively charged metal ions or proteins [30,31]. This means it is possible that PA can combine with wool fiber (natural protein fiber) by means of the electrostatic interaction between the positively charged amino groups in wool and the negatively phosphate groups in PA. In the work reported here, PA was applied to wool fabric through an exhaustion process with the aim of improving the flame resistance of woolen textiles. The important factors affecting the adsorption of PA such as pH, temperature and PA concentration were discussed. The combustion and thermal properties of the treated fabrics were evaluated via limiting oxygen index (LOI), vertical burning test, pyrolysis combustion flow calorimetry (PCFC) and thermogravimetry (TG). Scanning electron microscopy (SEM) coupled with energy dispersive X-ray spectroscopy (EDS) was used to investigate the morphology of the treated fibers. Furthermore, the phosphorus content of the unburned and burned wool fabrics was detected by inductively coupled plasma optical emission spectrometry (ICP-OES).

## 2. Materials and Methods

### 2.1. Materials

The scoured, woven wool fabric (warp and weft thread, 156 dtex × 2) for color fastness tests up to the standard GB/T 7568.1-2002 was purchased from Shanghai Textile Industry Institute (Shanghai, China) of Technical Supervision. Phytic acid (PA) (70% aqueous solution) of analytical reagent grade was obtained from the Chengdu Ai Keda Chemical Technology Co. Ltd., Chengdu, China. Sodium hydroxide of analytical reagent grade was purchased from Sinopharm Chemical Reagent Shanghai Co. Ltd., Shanghai, China.

### 2.2. Experiments of the Adsorption of PA

All of the adsorption and treatment experiments of PA were carried out in the sealed and conical flasks immersed in a XW-ZDR low-noise oscillated dyeing machine (Jiangsu Jingjiang Xingwang Dyeing and Finishing Machinery Factory, Jingjiang, China). The liquor ratio was 50:1. At the end of processing, the wool fabrics were washed in distilled water and then dried in the open air.

#### 2.2.1. Effect of pH on the Adsorption of PA

The pH of PA solutions was adjusted to 1.2, 2.1, 3.0 and 4.1 by addition of 1 M sodium hydroxide solution and detected by a PHS-3C pH meter (Shanghai REX Instrument Factory, Shanghai, China). The wool fabrics were immersed in the solutions of 120% owf (on the weight of fiber) PA at 30 °C, and the temperature was raised to 90 °C at a rate of 2 °C/min and the treatment continued for 60 min.

#### 2.2.2. Effect of Temperature on the Adsorption of PA

To assess the effect of immersing temperature on the adsorption of PA, wool fabrics were treated with the solutions of 120% owf PA for which the pH was adjusted to 1.2. The samples were immersed into the solutions at 30 °C, and the temperature was raised to different temperatures (50–98 °C) at a rate of 2 °C/min with a holding time of 60 min.

#### 2.2.3. Equilibrium Adsorption Isotherm of PA

The adsorption isotherm of PA on wool fabric was measured in a series of PA solutions of various concentrations (10%–200% owf) at 90 °C with the pH adjusted to 1.2. The isotherm was determined on the basis of the adsorption for 100 min as the tests showed that the equilibrium adsorption was reached in 60 min.

#### 2.2.4. Building-up Property of PA

The building-up property of PA on wool fabric was measured in the solutions (pH 1.2) of 10%–200% owf PA. The fabrics were immersed in the solutions at 30 °C, and subsequently the solutions were heated to 90 °C at a rate of 2 °C/min and the treatment continued for 60 min.

### 2.3. FR Treatment of Wool Fabric

In the adsorption experiments described above, a small-scale sample of 1 g and a high liquor ratio of 50:1 were used for the purposes of the convenience and accuracy of this study. However, the samples with large areas were needed for the investigations on the FR and physical properties of wool, so a sample of 8 g and a low liquor ratio of 15:1 were employed to carry out the FR treatment. The other processing parameters used this treatment were the same as those in Section 2.2.4. The samples so obtained were used for the flame retardancy research. In particular, Wool-0, Wool-40, Wool-80 and Wool-120 mentioned in the results and discussion section represent the fabrics treated with 0%, 40%, 80% and 120% owf PA, respectively, and their corresponding weight gain was 0%, 10.6%, 15.0% and 17.9%, respectively.

### 2.4. Measurements

#### 2.4.1. Adsorptions of PA

The absorption spectra and absorbance (λ_max_ = 261 nm) of PA solutions were measured using a Shimadzu UV-1800 UV–Vis spectrophotometer (Shimadzu Co., Kyoto, Japan). Using a previously established absorbance and concentration relationship at the λ_max_ of the PA solution, the quantity of PA in solution was able to be calculated, and the percentage of exhaustion was determined using Equation (1), where *m*_0_ and *m*_1_ are the quantities of PA in solution before and after treatment, respectively. The quantity of PA on wool was calculated by the difference in the initial and final concentrations of PA in solution.

(1) $$\text{Exhaustion }(\%)=100\times (m_{0}-m_{1})/m_{0}$$

#### 2.4.2. Weight Gain

The fabrics before and after treatment were dried in an oven at 60 °C for 30 min, and then weighed quickly. The weight gain of the treated fabric was calculated using Equation (2).

(2) $$\text{Weight gain }(\%)=100\times (W_{2}-W_{1})/W_{1}$$

where *W*_1_ and *W*_2_ are the weights of the fabrics before and after treatment, respectively.

#### 2.4.3. LOI Test

The LOI values of the fabrics were measured according to GB/T 5454-1997 (equivalent to ASTM Standard Method D2863) with the FTT0080 oxygen index apparatus (Fire Testing Technology Ltd., East Grinstead, UK).

#### 2.4.4. Vertical Burning Test

The vertical burning test was conducted according to GB/T 5455-2014 (equivalent to ASTM Standard Method D6413) with the YG 815B automatic vertical flammability cabinet (Ningbo Textile Instrument Factory, Ningbo, China). The burning behavior of the fabrics for the vertical burning test was classified according to GB 8624-2012.

#### 2.4.5. PCFC Test

The PCFC analysis was conducted using the FTT0001 microscale combustion calorimetry (Fire Testing Technology Ltd., East Grinstead, UK) according to ASTM Standard Method D7309. About 5 mg specimen was thermally decomposed in an oxygenated environment at a constant heating rate of 1 °C/s.

#### 2.4.6. TG Analysis

The thermogravimetric (TG) curves were recorded with the Diamond TG/DTA SII thermal analyzer (Perkin-Elmer, Waltham, MA, USA) from 30 to 700 °C at the scan rate of 10 °C/min under a flow of air (20 mL/min). Each sample was controlled to 4–5 mg in primary weight.

#### 2.4.7. FT-IR Spectra

The Fourier transform infrared (FT-IR) spectra of wool samples were measured by the Nicolet 5700 FT-IR spectrometer (Thermo Fisher Scientific Inc., Waltham, MA, USA) over the wavenumber range of 4000–400 cm^−1^ using KBr pellets. All of the IR data were collected from 32 scans with a resolution of 4.0 cm^−1^.

#### 2.4.8. SEM Observation

The wool fibers as well as the chars obtained in the PCFC experiments were first sputter-coated with a gold layer, and then their surface morphologies were observed by a TM3030 tabletop scanning electron microscope (Hitachi High Technologies America, Inc., Schaumburg, IL, USA) with a 15 kV accelerated voltage. The phosphorus content was tested by EDS with a 15 kV electron beam energy.

#### 2.4.9. ICP-OES

The phosphorus content of the unburned and burned wool fabrics was measured as follows: the dried fabric and char residue (0.1 g) were digested in 30 mL of 15 wt % nitric acid for 2 h at 80 °C. A phosphorus standard solution was used for calibration, and the digestion solutions were diluted to be in the calibration range prior to analysis. ICP-OES was conducted on the ICAP 6300 DUO (Thermo Fisher Scientific Inc., Waltham, MA, USA) with argon plasma at the wavelength of 178.284 nm. The P content was calculated by Equation (3).

(3) $$\text{P content }(\text{mg}/\text{g})=\frac{C_{s}}{W}\times V$$

where *C*_s_ is the P concentration in the digestion solution analyzed by ICP-OES (mg/L); *V* is the volume of the digestion solution (0.03 L); *W* is the weight of the dried sample (0.1 g).

#### 2.4.10. Whiteness Index

The whiteness of wool fabrics was measured with the WSB-2 digital whiteness meter (Shanghai Pingxuan Scientific Instrument Co. Ltd., Shanghai, China) in accordance with ASTM Standard Method E313. Each sample was tested four times at different positions and the average of the data was used.

#### 2.4.11. Mechanical Performance

The tensile strength of wool fabrics was measured according to ISO 13934-1-2013 with the Instron 3365 tester (Illinois Tool Works Inc., HighWycombe, Buckinghamshire, UK). For each sample, five test specimens were tested and the stress-strain plot that represents the average result was reported. The samples were conditioned under a standard atmospheric condition (65% ± 5% relative humidity and 21 ± 1 °C) for 24 h before testing.

#### 2.4.12. Durability to Washing

The washing test of the treated wool fabric was carried out in the pots housed in a WashTec–P fastness tester (Roaches International, WestYorkshire, UK). The washing solution contained 4 g/L commercial detergent, and the liquor ratio was 50:1. Each wash was conducted at 40 °C for 5 min. After one wash, the fabric was removed, gently squeezed, and rinsed with tap water. Then repeated washing was carried out until a total of 250 min was reached.

## 3. Results and Discussion

### 3.1. Adsorption Properties of PA

#### 3.1.1. Effect of pH on the Uptake of PA

**Figure 1:**
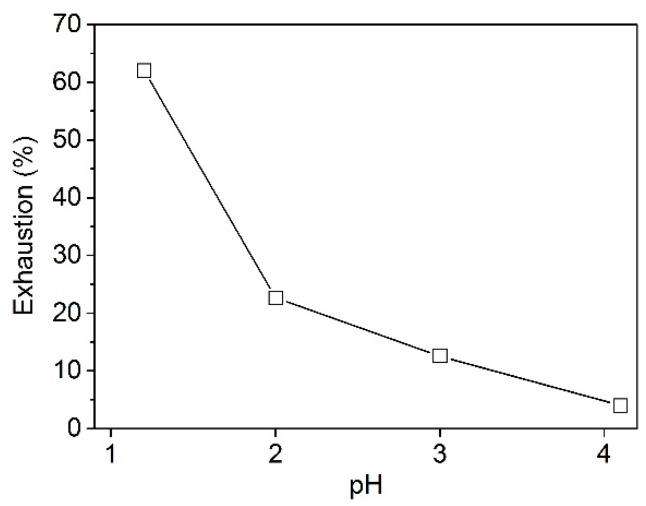
Effect of pH on the uptake of PA by wool.

The exhaustion of PA at different pH values was tested and the results are shown in Figure 1. The adsorption of PA on wool fiber was found to be sensitive to pH. The exhaustion decreased obviously with increasing pH in the range of 1.2–4.1. This indicates that the electrostatic interactions between the positively charged amino groups in wool fiber and the anionic phosphate groups in PA exert an important role in the adsorption of PA on wool. The higher extent of adsorption at pH 1.2 is due to the greater protonation extent of amino groups which gives rise to ion-ion interaction. As the quantity of the protonated amino groups in wool fiber was greatly reduced at pH 4.1, the exhaustion of PA on wool fiber became quite low. This implies that non-electrostatic interactions between PA and wool fiber are fairly small.

#### 3.1.2. Effect of Temperature on the Uptake of PA

**Figure 2:**
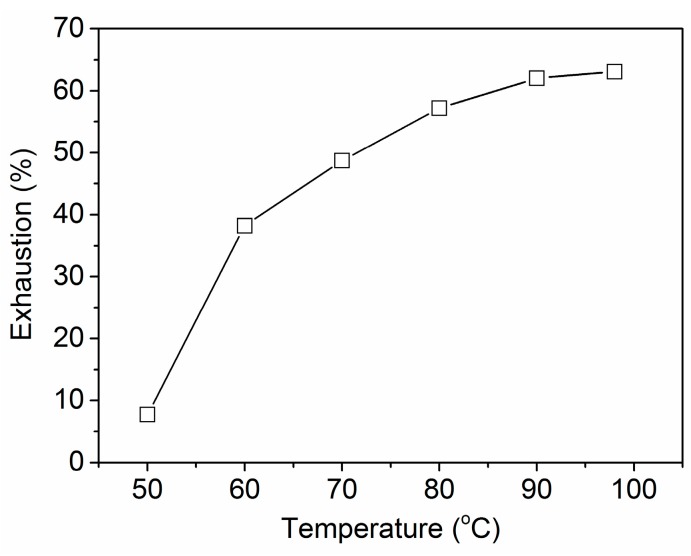
Effect of temperature on the uptake of PA by wool.

Wool is a natural protein fiber and its surface is covered by cuticles which can reduce the rate of small molecules (e.g., dyes) entering the fiber interior. Because a high temperature can increase the swelling extent of wool fiber, temperature is an important parameter during the wet processing of wool. The uptake of PA by wool at different temperatures is shown in Figure 2. A small exhaustion was found at 50 °C, this being attributed to the surface barrier action of the cuticle layer to PA penetration into fiber interior. The exhaustion increased with increasing temperature because high temperature can increase the swelling degree of wool fiber and thus promote the diffusion of PA into fiber interior. As a result, an immersing temperature of 90 °C was needed to ensure the higher adsorption of PA by wool.

#### 3.1.3. Equilibrium Adsorption Isotherm of PA

**Figure 3:**
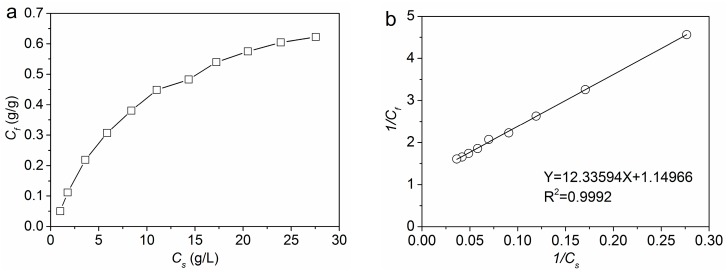
Adsorption isotherm of PA on wool: (**a**) plot of *C*_f_ *vs. C*_s_ and (**b**) plot of 1/*C*_f_ *vs.* 1/*C*_s_.

The equilibrium adsorption isotherm of PA on wool fiber can be depicted by the plot of the concentration of PA on wool fiber (*C*_f_, g/g) as a function of the concentration of PA in solution (*C*_s_, g/L) at equilibrium. The effect of initial PA concentration on equilibrium adsorption was evaluated within the range of 10%–200% owf. As shown in Figure 3a, with an increase in the initial concentration of PA, there was an increase in adsorption capacity until equilibrium was attained.

The experimental data were fitted to the following Langmiur equation:

(4) $$C_{f}=\frac{SKC_{s}}{1+KC_{s}}$$

where *S* is the saturation concentration of PA on wool fiber, and K is the Langmuir affinity constant.

The Langmuir isotherm plot of 1/*C*_f_ *vs.* 1/*C*_s_ is shown in Figure 3b. The Langmuir equation exhibited a high correlation (*R*^2^ = 0.9992) to the experimental data, indicating that the Langmuir isotherm is an appropriate model to describe the adsorption behavior of PA on wool fiber. The saturation of PA adsorption on wool fiber was 0.8792 g/g and the Langmuir affinity constant was 0.0932 L/g. It can therefore be concluded that electrostatic interactions occur between the anionic PA molecules and the protonated amino groups in wool fiber.

#### 3.1.4. Building-up Property of PA

**Figure 4:**
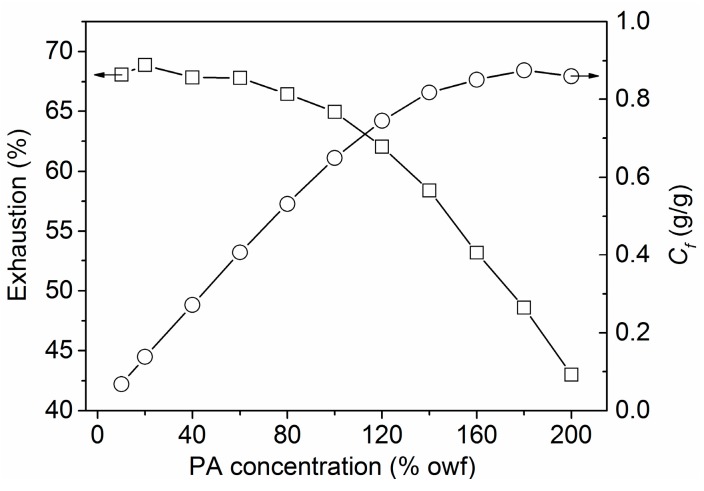
Influence of initial PA concentration on its uptake by wool.

The building-up property of PA depends on its affinity to wool fiber and its adsorption saturation, which is of great importance for practical application. Considering practical application conditions, the building-up property of PA was determined in a temperature-rise process in place of a constant temperature employed for the adsorption isotherm studies. The building-up property of PA expressed by the exhaustion as well as the quantity of PA absorbed by wool (*C*_f_) is depicted in Figure 4. The quantity of adsorption linearly increased with increasing PA concentration in the range of 10%–140% owf, indicating a good building-up property of PA on wool fiber. Figure 4 also reveals that the lower exhaustion was obtained as the application concentration of PA increased, but the exhaustion was still higher than 60% at a PA dosage of 120% owf. The results indicate that PA has a fairly high utilization rate.

### 3.2. Flame Retardancy

#### 3.2.1. Flammability Test

**Figure 5:**
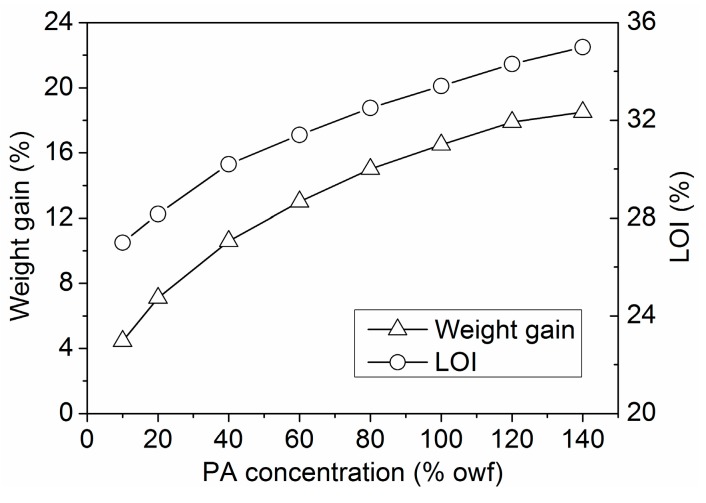
Weight gain and LOI of the wool fabrics treated with PA.

LOI and vertical burning tests are widely used to determine the relative flammability of polymeric materials. The LOI and weight gain of the wool fabrics treated with PA solutions of different concentrations are shown in Figure 5. Clearly, the higher weight gain and LOI of the fabrics were obtained when a higher PA concentration was used. The flame retardancy of the treated wool fabrics also showed a linear relationship with the content of PA on fiber substrate. The untreated wool fabric had a low LOI of 23.6%. When treated with 140% owf PA, the wool fabric experienced a weight gain of 17.9% and exhibited a very high LOI of 35.2%.

**Figure 6:**
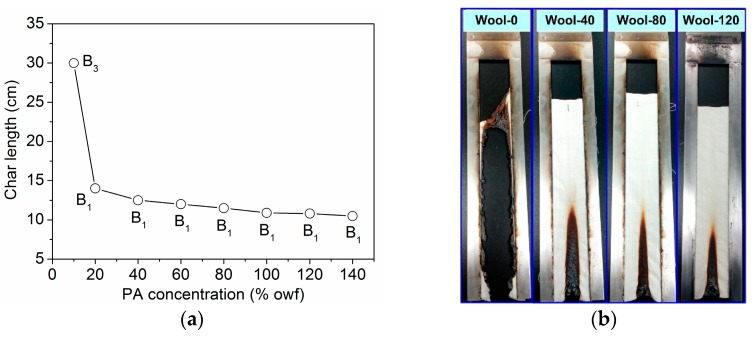
Char length (**a**) and photographs (**b**) of the treated wool fabrics after vertical burning tests.

There are three different classifications of FR fabrics based on the vertical burning test according to GB 8624-2012: (1) B_1_ classification: char length ≤15 cm, after flame time ≤5 s, after glow time ≤15 s; (2) B_2_ classification: char length ≤20 cm, after flame time ≤15 s, after glow time ≤30 s; and (3) B_3_ classification: no special requirement. The untreated wool fabric had a very high combustion speed, and it was completely burned out. Figure 6a shows the char length of the wool fabrics treated with PA, and Figure 6b displays the photographs of the burned fabrics whose char length approaches the calculated average value. PA could effectively strengthen the char formation ability of wool fabric. The inflated char could successfully withstand the open fire below and prevent neighboring polymeric substrate from further attack by heat flux, so the treated wool fabrics with good flame retardancy are self-extinguished after the removal of ignition source. As shown in Figure 6, the char length of the fabrics treated with PA decreased obviously. More than 20% owf of PA could endow wool fabric with a B_1_ classification according to the vertical burning test. The aforementioned results indicate that PA can significantly improve the flame retardancy of wool fabric.

#### 3.2.2. PCFC Analyses

As a supplement to LOI and vertical burning tests, the PCFC test is an effective analytical technique for measuring the flammability of milligram-sized materials and screening new FR agents [32]. Although PCFC as a milligram-scale test cannot replace flame and fire tests, it has been shown to be correlated with the data from some fire tests [32]. So the PCFC test was employed to evaluate the FR behavior of the treated wool fabric by measuring the heat release rate (HRR), peak heat release rate (pHRR in W/g), heat release capacity (HRC in J/(g K)), total heat release (THR in kJ/g) and temperature at maximum heat release rate (*T*_max_).

**Figure 7:**
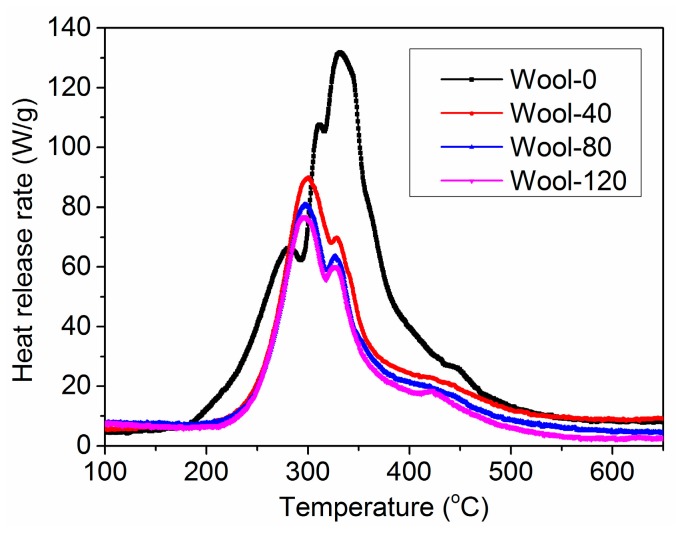
HRR curves of the wool fabrics from the PCFC test.

The pHRR is an important parameter to evaluate the intensity of combustion. Figure 7 shows the HRR curves of the wool fabrics treated with PA. The corresponding combustion data are listed in Table 1. It is evident that the pHRR values of the treated wool fabrics were much lower than that of pure wool fabric, and decreased with increasing PA concentration. The untreated fabric had a high pHRR value of 131.7 W/g. As for Wool-40, Wool-80 and Wool-120, the pHRR decreased to 89.8, 80.7 and 76.7 W/g, which were 31.8%, 38.7% and 41.7% reduction, respectively. HRC is a relatively good predictor of heat release rate in flaming combustion. As shown in Table 1, the treated wool fabrics had much lower HRC values than pure wool, and the HRC value decreased further with increasing PA concentration. THR calculated from the total area under the HRR peaks is another important parameter for fire hazard evaluation [33]. THR showed the same trend as pHRR and HRC. In addition, the *T*_max_ values of the treated wool fabrics decreased compared to pure wool possibly because of the lower initial thermal stability of PA. The PCFC data presented here convincingly demonstrate that the treated wool fabrics possess low flammability, indicating the effectiveness of PA as a FR agent on wool fiber.

From the PCFC test, the information about the charring of wool fabrics can also be obtained. The morphological structures and P content of the char residues obtained in this test were discussed in Section 3.2.5 and Section 3.2.6.

#### 3.2.3. TG Analyses

**Figure 8:**
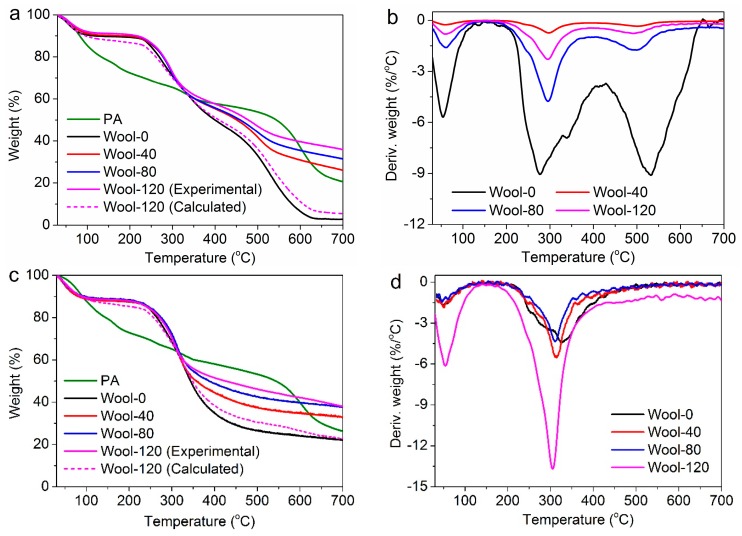
TG and DTG curves of wool fabrics under air (**a**,**b**) and nitrogen (**c**,**d**).

The thermogravimetric (TG) and derivative theremogravimetric (DTG) curves under air and nitrogen were used to study the thermal degradation and stability of wool fabrics. The TG and DTG curves of the samples and the calculated TG curve of Wool-120 are shown in Figure 8. The related thermal decomposition data are listed in Table 2, where *T*_20%_ and *T*_50%_ are defined as the temperatures corresponding to a weight loss of 20% and 50%, respectively.

As stated in previous reports, three processes take place during the wool pyrolysis process under air [9,10,12]. From Figure 8b, the first process was observed from 30 to 160 °C, corresponding with the desorption of water physically bound to wool fiber and the dehydration of wool fiber. The second process occurred from 190 to 425 °C, coinciding with the temperature range over which a number of defined pyrolysis reactions take place in wool. At the second stage, the hydrogen-bond peptide helical structure ruptures and the ordered regions of wool fiber undergo a solid-to-liquid phase change; also, the cleavage of the disulphide bonds occurs and a number of volatiles including hydrogen sulfide and sulphur dioxide are released. The third process is dominated by the char oxidation reactions. At this process, the char and remaining hydrocarbon species were further oxidized to carbon monoxide and carbon dioxide. However, Figure 8c,d show that the thermal degradation of wool under nitrogen proceeded only in two steps, during which the further char oxidation process might be not involved.

Compared to wool, PA exhibited higher weight loss at low temperature, and lower weight loss at high temperature. The adsorbed PA by wool is responsible for an obvious increase in the decomposition temperature of wool, as revealed by the *T*_20%_ and *T*_50%_ in Table 2 and well demonstrated by Figure 8. This indicates that the introduction of PA changes the decomposition behavior of wool, and enhances the thermal stability of wool. Assuming that no chemical or physical interactions between wool and PA occur, the calculated TG curve of Wool-120 was able to be obtained on the basis of the simply additive contribution of wool and PA. The experimental and calculated TG curves of Wool-120 did not overlap as shown in Figure 8a,c, and the experimental curve exhibited much higher residues compared to the calculated one. This observation reveals that some interactions between wool and PA chains take place during the heating, and the degradation of PA interferes with the degradation reactions of wool. Compared to 2.8% char residue for Wool-0 at 700 °C under air, the char residues of Wool-40, Wool-80 and Wool-120 were 26.1%, 31.5% and 36.0%, respectively. The similar trend for char residues was found in the case of nitrogen atmosphere. Such high char residues of the FR fabrics indicate the high thermal insulation of the char and the good resistance of char to the aforementioned oxidation reactions. This also implies that the FR mechanism of the treated wool involves a significant condensed-phase activity.

#### 3.2.4. FT-IR Analyses

**Figure 9:**
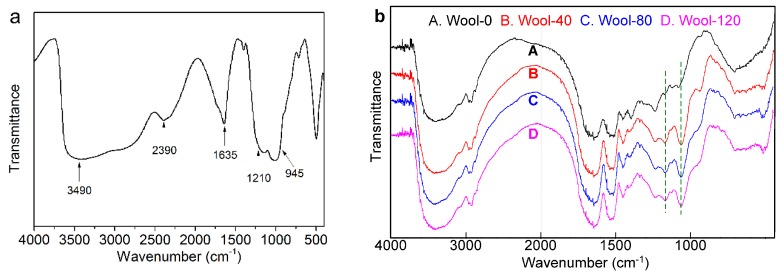
FT-IR spectra of (**a**) PA and (**b**) wool fabrics.

The FT-IR spectrum of PA is shown in Figure 9a. The strong peak around 3490 cm^−1^ belongs to the OH stretching vibration of PA. The peak at 1635 cm^−1^ is caused by the hydration of water molecules [34]. The broad peak between 1210 and 945 cm^−1^ is associated with the stretching vibration of P=O, O–P–C, P–O and O–P–O structures [34,35,36]. The FT-IR spectra of the treated and untreated wool fabrics are shown in Figure 9b. The peaks around 1635 and 1520 cm^−1^ are the characteristic bands of amide I (C=O stretching) and amide II (N–H bending and C–H stretching) for wool fiber, respectively. The characteristics of the amide I band are associated with the C=O stretching of α-helix, β-sheet and random disorder secondary structures of keratin [37]. In the spectrum of the treated wool, the new peaks at 1168 and 1060 cm^−1^ are due to the stretching vibration of P=O and O–P–C structure [34,35,36], respectively. These results showed that the treated wool fibers contain P=O and P–O–C structures, which are helpful to improve the flame retardancy of the fabric.

#### 3.2.5. SEM Analyses

**Figure 10:**
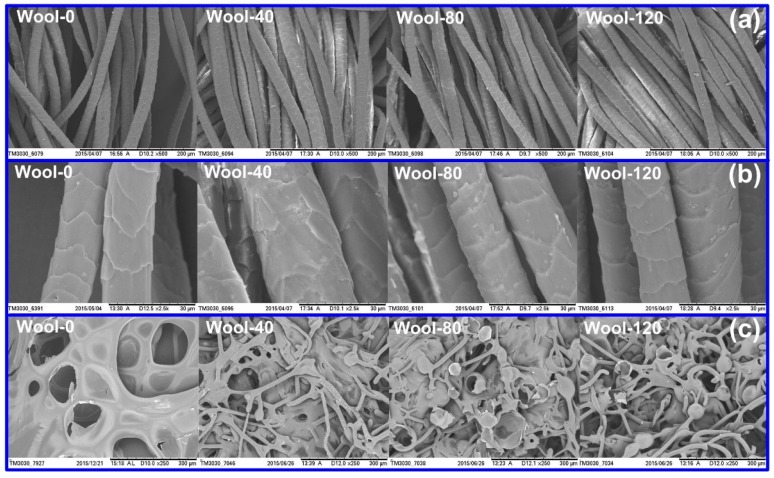
SEM micrographs of wool fibers (**a**,**b**) and char residues (**c**).

SEM was employed to study the morphological structures of wool fibers and char residues. Figure 10 shows the SEM micrographs of the wool fibers and corresponding char residues after the PCFC test. The untreated wool displayed the clean scales on its surface. The low magnification micrographs (Figure 10a) showed that PA did not accumulate within the fabric texture. From the high magnification micrographs (Figure 10b), a handful of particles were found to be attached to the surfaces of the treated wool fibers. After the FR treatment, most loose chemicals on the fiber surface should be washed off by the rinsing process. Only a handful of PA depositions on the fiber surface prove that most of the adsorbed PA molecules have completely diffused into the interior of wool fiber.

From Figure 10c, a porous and fragile char was observed for the untreated wool due to the complete burning and insufficient char formation during the combustion. A series of voids are possibly caused by the volatiles which inflate the char. In the case of the treated wool, during the combustion, the original fiber structure was destroyed, but PA led to the formation of some additional fibrous chars which were not seen in the control sample. These observations indicate that the rate of char oxidation was decreased due to the presence of PA, and the char was much more complex [13]. In addition, unbroken intumescent-like bubbles were also found on the char residues of the treated wool fibers. These intumescent-like bubbles can hide combustible or volatile gases, slow down the heat transfer between gas and condensed phases, and prevent the underlying polymeric substrate from further attack by heat flux in a flame [38,39], resulting in the good FR performance of the treated wool fabric.

#### 3.2.6. P Content Determination

**Figure 11:**
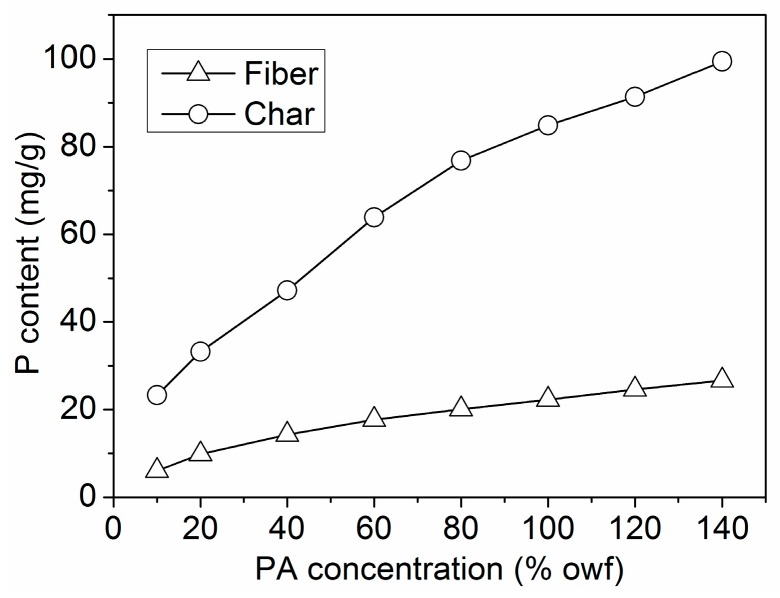
P content of wool fibers and corresponding char residues determined using ICP-OES.

**Figure 12:**
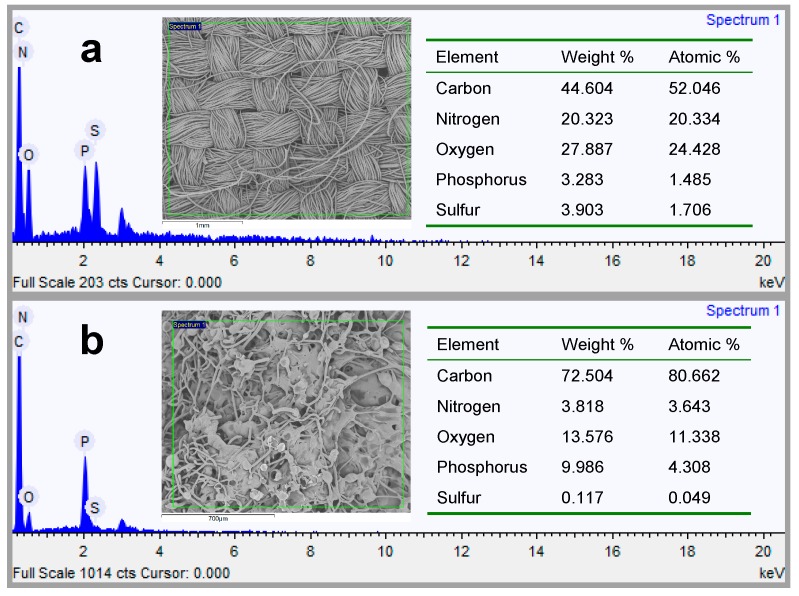
SEM-EDS micrographs of wool fiber (**a**) and char residue (**b**).

**Figure 13:**
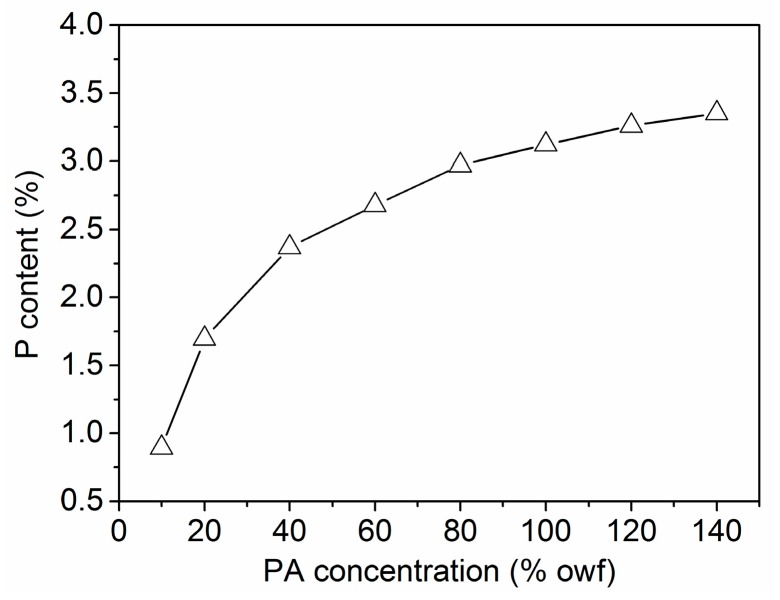
P content of wool fibers determined using SEM-EDS.

The total P content of the treated wool fibers and corresponding char residues was determined by ICP-OES, and the relative data are shown in Figure 11. The P content of the wool fibers and char residues increased with increasing PA concentration. Obviously, the P content of the char residues was much higher than that of the wool fibers. In addition, SEM-EDS was also used to evaluate the content of element P on the surface of the treated wool fibers and the residues after the PCFC test. The SEM-EDS results of the wool fabric treated by 120% owf PA and the corresponding char residue are depicted in Figure 12. From Figure 13, it is clear that the P content of the wool fiber surface increased with increasing PA concentration. The P content of the unburned Wool-40, Wool-80 and Wool-120 was 2.38, 2.97 and 3.13 wt %, respectively, while that of the corresponding char residues was 5.88, 7.96 and 10.16 wt %, respectively. These results indicate that the element P participates in the formation of the char layer, which hinders the transfer of heat flow and combustible gas and thus provides good flame retardancy. Therefore, it can be concluded that the condensed-phase mechanism is applicable to the FR wool fabric treated with PA.

### 3.3. Physical Properties

#### 3.3.1. Whiteness Index

**Figure 14:**
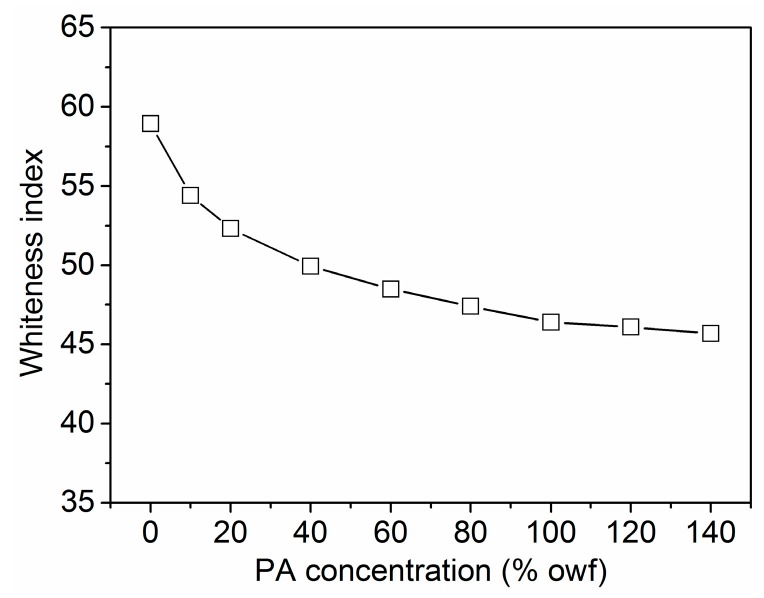
Whiteness of the wool fabrics treated with PA.

The whiteness indexes of wool fabrics are shown in Figure 14. It can be observed that the application of PA reduced the whiteness of the fabrics, and the whiteness indexes decreased with increasing PA concentration. PA used in the present study is a slightly yellow solution, and the inherent yellow color of PA may induce the yellowing of wool fabrics. In addition, the oxidation yellowing of PA under heating [40] as well as the hydrolysis of the peptide chains of wool fiber at a low pH used in the PA treatment can also contribute to the yellowing of wool fabrics.

#### 3.3.2. Mechanical Performance

**Figure 15:**
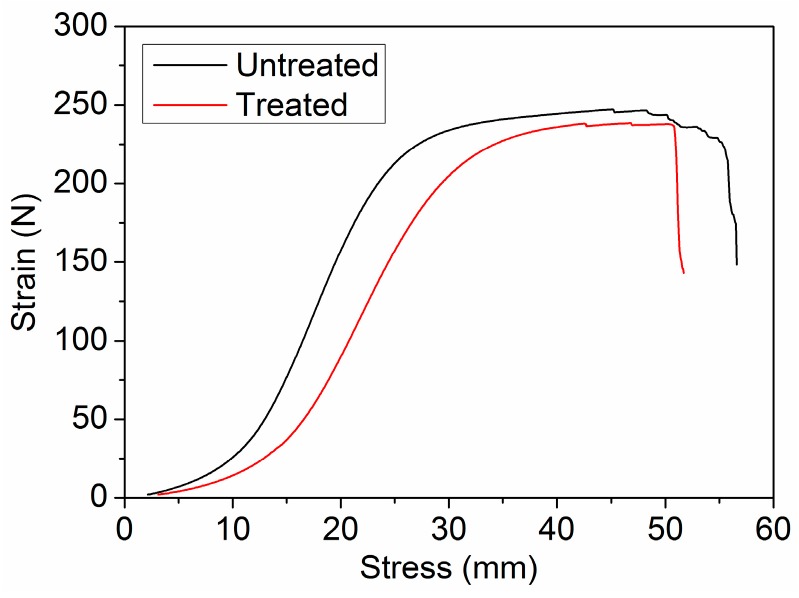
Stress-strain curves of the untreated and treated wool fabrics.

The tensile strength of the treated wool fabric was compared to that of the untreated wool fabric to see whether PA has any adverse effect on mechanical performance. Tensile strength evaluation was obtained by measuring the maximum stress of stress-strain plot when the fabric starts to break. The stress–strain plots representing average values are shown in Figure 15. PA could combine with the wool fiber, and hydrolyze the amide linkage of the peptide chains at a low pH. This means the treated wool (Wool-120) had a loss of tensile strength and elongation at break compared to the untreated wool. The original fabric had a mean tensile strength of 245.9 ± 1.0 N and elongation of 22.1% ± 1.1% at break, whereas the mean tensile strength and elongation of the treated fabric were 237.4 ± 3.6 N and 21.0% ± 1.8%, respectively. PA treatment produced slightly decreased strength and elongation. Such a small decrease can be acceptable in the wet processing of wool fabric.

### 3.4. Durability of FR Effect

**Figure 16:**
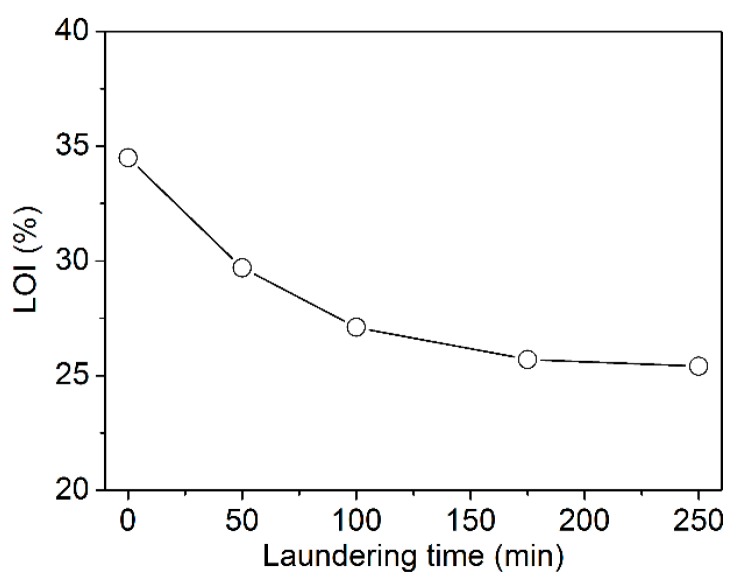
LOI of the treated wool fabric after laundering.

The washing resistance of the FR effect of textiles is directly related to the applicability of the corresponding products. Based on this, the treated fabric (Wool-120) was subjected to washing for different times. The result is shown in Figure 16. The FR performance of the treated fabric expressed by the LOI value decreased with increasing washing duration due to the water solubility of PA. However, PA can combine with wool fiber due to the electrostatic interaction between the positively charged amino groups in wool and the negatively charged phosphate groups in PA. Consequently, the LOI value of the treated fabric was still higher than 27.0% after laundering for 100 min. All the same, special measures should be taken to improve the wash resistance of the FR wool fabrics in the future work.

## 4. Conclusions

In the previous studies, PA as an anionic compound together with several cationic compounds containing silicon or nitrogen was used to improve the FR performance of cellulose-based materials. In this study, PA acted as an individual FR agent and was successfully applied to improve the FR properties of wool fabric using an exhaustion technique. The equilibrium adsorption isotherm of PA on wool could be described by the Langmuir model due to the electrostatic interactions between PA and wool fiber. The flame retardancy of the treated wool fabrics was confirmed by the results from the LOI, vertical burning and PCFC tests. PA has been proven to be a potential FR agent because of its high char-forming ability. The P content of the treated wool fibers was much lower than that of the corresponding char residues, indicating that P participates in the formation of the char layer, and the condensed-phase mechanism is suitable for FR wool fabrics. The use of PA provides an opportunity for producing FR wool fabrics using a green FR agent. In future work, some measures should be taken to improve the wash resistance of FR wool fabrics.

## Figures and Tables

**Table 1:** PCFC parameters for the wool fabrics treated with PA.

Samples	HRC (J/(g K))	pHRR (W/g)	THR (kJ/g)	*T*_max_ (°C)
Wool-0	130 ± 4	131.7 ± 3.5	14.0 ± 0.5	331.7 ± 3.4
Wool-40	85 ± 1	89.8 ± 1.9	8.2 ± 0.1	300.3 ± 0.5
Wool-80	78 ± 2	80.7 ± 0.4	7.4 ± 0.2	296.6 ± 0.5
Wool-120	74 ± 2	76.7 ± 1.6	6.7 ± 0.2	296.1 ± 2.7

**Table 2:** TG data of wool fabrics under air and nitrogen.

Samples		*T*_20%_ (°C)	*T*_50%_ (°C)	Char Residues at 700 °C (%)	
				Experimental	Calculated
Air	PA	132.9	543.8	20.6	–
	Wool-0	270.0	399.9	2.8	2.8
	Wool-40	278.3	453.9	26.1	4.6
	Wool-80	277.8	459.6	31.5	5.1
	Wool-120	279.8	478.1	36.0	5.5
Nitrogen	PA	145.4	543.2	26.3	–
	Wool-0	264.9	343.7	22.3	22.3
	Wool-40	270.6	357.6	33.2	23.7
	Wool-80	274.8	386.9	37.6	24.2
	Wool-120	271.5	425.9	38.0	24.5

## Abbreviations

The following abbreviations are used in this manuscript:

DTG: Derivative Theremogravimetric
EDS: Energy Dispersive X-ray Spectroscopy
FR: Flame Retardant
FT-IR: Fourier Transform Infrared
ICP-OES: Inductively Coupled Plasma Optical Emission Spectrometry
LOI: Limiting Oxygen Index
PCFC: Pyrolysis Combustion Flow Calorimetry
PA: Phytic Acid
SEM: Scanning Electron Microscopy
TG: Thermogravimetry
